# Chair of Chemistry and Pharmacy in the Atlanta Medical College

**Published:** 1858-02

**Authors:** 


### Chair of Chemistry and Pharmacy in the Atlanta Medical College.

We are enabled to state that the chair of Chemistry and Pharmacy in the Atlanta Medical College will continue to be occupied by Prof. Alexander Means, M. D., the distinguished gentleman who has heretofore held the position.

In this connection, it is perhaps proper to state that for a time it seemed probable his services would be lost to the Institution, his resignation having been placed before the Faculty, though not acted upon by the Trustees. Previous to the selection of an individual to fill the expected vacancy, it being understood that he desired to resume his connection with the College, he was allowed to withdraw his resignation by the unanimous consent of the Faculty, and will, we are assured, bring renewed energy to the discharge of the duties of his Chair. Those who have announced the resignation, will of course make the correction.

